# The Impact of Sleep and Circadian Disturbance on Hormones and Metabolism

**DOI:** 10.1155/2015/591729

**Published:** 2015-03-11

**Authors:** Tae Won Kim, Jong-Hyun Jeong, Seung-Chul Hong

**Affiliations:** Department of Psychiatry, College of Medicine, The Catholic University of Korea, Suwon, Seoul 442723, Republic of Korea

## Abstract

The levels of several hormones fluctuate according to the light and dark cycle and are also affected by sleep, feeding, and general behavior. The regulation and metabolism of several hormones are influenced by interactions between the effects of sleep and the intrinsic circadian system; growth hormone, melatonin, cortisol, leptin, and ghrelin levels are highly correlated with sleep and circadian rhythmicity. There are also endogenous circadian mechanisms that serve to regulate glucose metabolism and similar rhythms pertaining to lipid metabolism, regulated through the actions of various clock genes. Sleep disturbance, which negatively impacts hormonal rhythms and metabolism, is also associated with obesity, insulin insensitivity, diabetes, hormonal imbalance, and appetite dysregulation. Circadian disruption, typically induced by shift work, may negatively impact health due to impaired glucose and lipid homeostasis, reversed melatonin and cortisol rhythms, and loss of clock gene rhythmicity.

## 1. Introduction

Human beings sleep for approximately one-third of their lifetime, but the endogenous mechanisms underlying sleep and its role in homeostasis remain to be fully elucidated. The circadian clock is an autonomous mechanism that prepares an organism to interact with external stimuli on cell, organ, and organism levels, according to a transcription-translation feedback loop [1]. The circadian system is characterized by an endogenous rhythmicity (i.e., independent oscillation) and an ability to shift its timing in accordance with external factors. The suprachiasmatic nucleus (SCN), located in the anterior hypothalamus above the optic chiasm, constitutes the major site of circadian rhythm regulation. Neuronal firing within the SCN propagates circadian rhythms and is also involved in coordinating the peripheral clock system. In addition to the circadian timing system, sleep stage, arousal level, rapid eye movement (REM), and slow-wave sleep are other important factors in circadian rhythms. The Process S and Process C models represent attempts to delineate the mechanism underlying sleep regulation [2]. In the Process S model, a homeostatic drive for sleep increases during waking and decreases during sleep. The Process C model refers to a propensity for circadian modulation during sleep. The interaction of the processes described by two-process model determines sleep quality and duration and arousal and performance levels. The levels of several hormones fluctuate according to the light and dark cycle and are also affected by sleep, feeding, and general behavior. The regulation of these hormones is influenced by interactions between the effects of sleep and the intrinsic circadian system such that adverse health effects due to hormonal or metabolic imbalances may occur when the sleep cycle and intrinsic timing system are unsynchronized. In this review, we discuss the association between sleep, metabolism, and the levels of various hormones, particularly in terms of the effects of sleep disturbance and circadian disruption on hormonal and metabolic function.

## 2. Sleep and Hormones

Several hormones are involved in sleep and circadian rhythmicity.

Growth hormone levels are increased during sleep and peak immediately subsequent to sleep onset [3, 4]. In a previous study, growth hormone levels, measured every 30 s during sleep, increased significantly during slow-wave sleep (SWS) compared with stages 1 and 2 and REM sleep [5]. Growth hormone is intermittently secreted during sleep, which could relate to the cyclic nature of SWS [6]. Posttraumatic stress disorder patients characterized by frequently disturbed sleep exhibited lower nighttime growth hormone plasma levels compared with healthy subjects [7]. Growth hormone replacement therapy, for growth hormone-deficient pediatric patients, enhanced EEG slow oscillation [8].

Melatonin exhibits robust circadian rhythmicity. Studies using constant routine and forced desynchrony protocols demonstrate that melatonin levels are high during the biological night versus day [9, 10]. The melatonin secretion pathway projects from the SCN to the paraventricular nucleus (PVN) and on to the upper thoracic spinal cord, superior cervical ganglion, and pineal gland [11]. Melatonin plays an important role in regulating human sleep. Administration of sustained-release or transdermal formulation melatonin reduces sleep latency, increases total sleep time, and improves sleep maintenance [12, 13]. Melatonin administration increases sleep spindle frequency on EEG [13]. Beta-blockers possess melatonin-suppressing properties; in patients taking atenolol in conjunction with melatonin, total wake time and sleep were improved [14]. In a study using subjects with cervical spinal cord injury and impaired melatonin production, sleep efficiency was improved compared with a control group with normal melatonin levels [15]. In another study, the average sleep efficiency of healthy subjects administered exogenous melatonin was increased by 88% during the circadian night, at which time endogenous melatonin was present. Melatonin did not affect sleep initiation or core body temperature. The efficacy of melatonin perseverated across the study and did not significantly affect the proportion of SWS or REM sleep [16]. Melatonin also confers a chronobiotic effect and can facilitate maintenance of an optimal sleep wake cycle [17, 18]. Blind subjects with free-running circadian rhythm disorder were entrained to a 24 h rhythm following melatonin administration.

Using a constant routine protocol, thyroid-stimulating hormone (TSH) concentrations reached their maximum and minimum in the middle of the biological night and biological afternoon, respectively [19, 20]. Total triiodothyronine (T3) and thyroxine (T4) concentrations were not associated with circadian rhythmicity [19]. A negative correlation between TSH levels and SWS has been reported [21, 22].

Cortisol exhibits circadian rhythmicity; its level rises rapidly in the middle of the biological night and peaks during the biological morning [23, 24]. Cortisol is released in a pulsatile manner throughout the 24 h with a circhoral ultradian rhythm. The pulsatile secretion of gonadotropin releasing hormone prevents the receptor desensitization [25, 26]. The SCN is at the center of this rhythm regulation spectrum. The hormonal pathway underlying this regulation projects from the SCN to the sub-PVN and dorsomedial nucleus of the hypothalamus (DMH) and then projects to the medial parvocellular part of the PVN, which stimulates corticotropin releasing hormone (CRH) [27]. The neuronal pathway involved in cortisol regulation projects from the SCN to the PVN and then to the adrenal cortex through the spinal cord [27]. Cortisol levels are reduced during SWS; a temporal relationship between SWS and decreased cortisol levels has also been reported. Intravenous infusion of cortisol increased SWS and decreased REM sleep; concerning the mechanism underlying this effect, Steiger reported that cortisol infusion suppresses CRH, thereby decreasing SWS in accordance with a negative feedback mechanism [28].

Ghrelin and leptin promote and suppress food intake, respectively [29, 30]. Ghrelin levels increase prior to habitual meal times and decrease thereafter [31, 32]. Several studies have evaluated the relationship between sleep and hormone levels [24]. Increased growth hormone levels and proportion of SWS and decreased REM sleep were observed following intravenous injection of ghrelin [33]. In a rodent study, SWS increased and REM sleep decreased following leptin infusion [34]. Elderly males administered ghrelin were subsequently characterized by an increased proportion of stage 2 and SWS sleep and decreased stage 1 and REM sleep [35]. Increased ghrelin levels during early-stage sleep and a blunted ghrelin response during sleep deprivation were also reported [36]. However, in another study no significant relationship between ghrelin levels and sleep stage was reported [37]. Concerning leptin, in one study, levels were increased during the biological night and peaked during the biological morning [38]. But Scheer et al. reported no fluctuations in leptin levels according to circadian rhythms [24].

## 3. Circadian Regulation of Carbohydrate

Daily oscillations in glucose metabolism have been consistently reported. Glucose utilization increases commensurate with physical activity and is greater during waking versus sleep. Evidence suggests that other factors may also be associated with oscillations in glucose metabolism, including circadian regulatory mechanisms. Suprachiasmatic nucleus-lesioned rats did not exhibit 24 h rhythmic variations in basal glucose concentrations [39]. In a recent systemic review, the SCN-PVN-autonomic nervous system axis played a critical role in the daily rhythms of hepatic glucose output [40]. Glucose homeostasis involves the coordination of exogenous (digestion and absorption) and endogenous (gluconeogenesis and utilization) mechanisms. The hepatocyte circadian clock is known to regulate glucose homeostasis. Several studies have investigated the genes associated with the cellular circadian rhythms involved in glucose metabolism. ClockΔ19 mutant mice are characterized by decreased oscillation in hepatic glycogen levels and glycogen synthase expression and activity [41]. In BMAL1 knockout mice, rhythmic expression of hepatic glucose regulatory genes such as PEPCK is absent, and exaggerated glucose clearance is observed [42]. Cryptochrome CRY1 and cryptochrome CRY2 are rhythmically expressed in the liver, which modulates hepatic gluconeogenesis. Elevated CRY1 expression during the night-day transition reduced fasting gluconeogenic gene expression commensurate with increased intracellular cAMP concentrations [43]. A relationship between melatonin and glucose metabolism has also been reported. Melatonin receptor knockout mice continue to express circadian PER1 and exhibit increased insulin secretion from the islets and altered insulin transcript circadian rhythms [44]. Another* in vivo* and* in vitro* study revealed that melatonin incubation enhanced glucagon expression and secretion; long-term oral administration of melatonin led to plasma glucagon elevation in rats [45].

## 4. Circadian Regulation of Lipid

Lipid metabolism also has daily rhythms. In rats, cholesterol and lipid absorption increase and decrease during high- (i.e., dark phase) and low-activity periods, respectively; such diurnal variation in lipid absorption is not observed in ClockΔ19 mutant mice [46]. Several different genes involved in lipid metabolism in the intestine, encoding apolipoprotein B (Apob), intestinal fatty acid binding protein (Fabp), and intestinal microsomal triglyceride transport protein (Mtp), exhibit circadian rhythms [47, 48]. Inhibition of clock and PER2 increased alcohol-induced intestinal hyperpermeability, which suggests a role for circadian genes in intestinal permeability regulation [49]. Circadian clock mutant mice exhibit low and nonrhythmic plasma levels of free fatty acids and glycerol, decreased lipolysis, and increased sensitivity to fasting. Circadian clock disruption promotes the accumulation of triglycerides in white adipose tissue and adipocyte hypertrophy [50]. Clock mutant mice showed hyperlipidemia, hepatic steatosis, hypertriglyceridemia, and hypercholesterolemia [51]. The daily oscillation of plasma triglyceride was disrupted in BMAL1 mutant mice [52]. BMAL1 also plays an important role in adipocyte differentiation and lipogenesis in rodents study [53]. BMAL1 mutant mice showed elevated respiratory quotient value, which indicated that BMAL1 was involved in the utilization of fat as an energy source [54]. Nocturnin (a clock-regulated deadenylase) knockout mice have reduced chylomicron transit into the plasma following the ingestion of dietary lipids [55].

## 5. Impact of Sleep Disturbance on Hormones and Metabolism

Increased food intake and decreased physical activity are both major factors in the development of obesity; epidemiological studies demonstrate that worldwide obesity prevalence continues to increase. Sleep duration might also be associated with obesity development [56]. Sleep debt in humans may increase obesity risk [57]. According to a poll by the National Sleep Foundation, the mean sleep duration of American adults was 6 h 40 min in 2008 compared with 8 h 30 min in 1960 [58]. Cross-sectional studies demonstrate a positive correlation between sleep deprivation and obesity risk [59, 60]. Several prospective studies provide strong evidence for a causal relationship between sleep deficit and obesity. In a UK study, shortened sleep duration in toddlers (<10.5 h/day) could increase obesity risk at 7 years of age [61]. Sugimori et al. evaluated sleep and body mass index (BMI) in pediatric patients at 3 and 6 years of age; <9 h of sleep was associated with increased obesity risk in males [62]. In a 5-year follow-up study, sleep deprivation was associated with a higher BMI 5 years later in then-adolescents [63]. Short sleep duration in childhood was associated with being overweight 3 years later [64]. In a longitudinal study, the relationship between sleep duration and long-term changes in visceral adiposity was investigated. Visceral adipose tissue (VAT) was assessed using computed tomography during the 6-year follow-up. Baseline short (<6 h/day) and long (>9 h/day) sleepers gained significantly more VAT; furthermore, changing from being a short to average sleeper protected against VAT gain [65]. These studies indicate that there is an association between sleep deprivation and obesity risk. In another study, sleep duration and dietary quality in adolescents were correlated; insufficient sleepers exhibited lower diet quality index scores compared with those sleeping for an optimal duration (≥9 h) [66].

Sleep deprivation is a risk factor for diabetes mellitus. An epidemiological study with an adult sample demonstrated an association between short sleep duration and diabetes mellitus risk [67]. Similarly, in a systemic review article, curtailed sleep duration was a risk factor for diabetes [68]. A laboratory study revealed an effect of sleep debt on metabolic and endocrine function [69]. Healthy young males were restricted to 4 h per night of bed time for six nights (sleep debt condition) followed by a seven-night 12 h bed time recovery period (sleep recovery condition). Glucose tolerance and thyrotropin concentrations were significantly lowered during sleep deprivation. Furthermore, evening cortisol concentration and sympathetic nervous system activity were increased during sleep deprivation, during which leptin levels were also at their lowest. The HOMA (homeostatic model assessment; insulin [mIU/L] ∗ glucose [mmol/L]/22.5) response was significantly higher in the debt versus recovery condition [70]. Increased HOMA levels are indicative of decreased glucose tolerance and/or insulin sensitivity. In a study comparing the effects of 4.5 and 8.5 h sleep conditions in healthy adults, phosphorylated Akt and total Akt response, which represent a critical step in the insulin-signaling pathway, were lowered during sleep deprivation [71]. The study also implied that sleep restriction resulted in insulin resistance at a cell-signaling level. The relationship between sleep duration and metabolic syndrome was explored in a Japanese study. Type 2 diabetes patients were divided into five groups according to sleep duration. Shorter and longer sleepers exhibited significantly more severe metabolic syndrome and other cardiovascular risk factors (U-shaped curve) [72]. To investigate the impact of sleep restriction on pediatric patients, a within-subjects, counterbalanced, crossover design was employed, with subjects increasing or decreasing time in bed by 1.5 h per night. In the increased sleep duration group food intake, fasting leptin levels, and bodyweight were all lowered [73]. In a sleep study using actigraphy, subjects slept for 1.4 h per night for 3 weeks, following which insulin sensitivity initially decreased and then recovered to baseline. Leptin concentration was reduced and bodyweight was unchanged [74]. Acute sleep restriction, for example, 4 h for 3 consecutive nights, reduced insulin sensitivity in healthy normal-weight adolescent males [75]. When adult subjects were restricted to two-thirds of their usual sleep time, their caloric intake was increased in the absence of alterations in energy expenditure or leptin and ghrelin concentrations [76]; 5 days of 4 hours of sleep was associated with increased glucose, insulin, cortisol, and leptin, decreased triglycerides, and no change in testosterone levels [77]. In another study, sleep restriction, to 4 h per night for 4 d, had no effect on glucose, insulin, or leptin profiles, with no evidence of increased insulin resistance [78].

In a randomized, crossover clinical study conducted by Spiegel et al., plasma leptin and ghrelin levels were measured and subjective hunger and appetite ratings during sleep deprivation and recovery obtained [57]. Subjects exhibited an 18% decrease in leptin (an anorexigenic hormone), 24% increase in ghrelin (an orexigenic hormone), 24% increase in hunger, and 23% increase in appetite when sleep was restricted to 4 h. Appetite for high carbohydrate food was increased by 32% during sleep deprivation; these data suggest that people will consume more calories when sleep-deprived due to increased hunger and decreased satiety. Another study explored the effects of sleep deprivation on energy intake. In a randomized crossover design, healthy volunteers slept for 5.5 or 8.5 h per night for 14 days [79]. Sleep-restricted subjects exhibited similar intake during regular meals but increased caloric consumption from snacks compared with the 8.5 h group. The average increase in snack-derived calories was approximately 220 kcal/day, suggesting that persistent sleep restriction could modify the amount, composition, and distribution of human food intake. Restriction to 6.5 h of bed time in adolescents was associated with increased consumption of high-calorie and glycemic index food [80]. The neuronal mechanisms underlying the effects of sleep restriction on food intake were investigated recently in a functional magnetic resonance imaging paradigm. Following five nights of 4 h bed time, healthy subjects were provided with healthy or unhealthy food during fasting. The response to unhealthy food stimuli was greater in brain reward and food-sensitive regions during sleep deprivation [81]. In another imaging study, sleep-deprived subjects exhibited decreased activity in appetite-sensitive regions of the frontal and insular cortices and increased amygdala activity during a food desirability rating task [82].

Even a single night of total sleep deprivation can influence energy expenditure and metabolism; in subjects with 24 h wakefulness, resting and postprandial energy expenditure were decreased; morning plasma ghrelin, nocturnal and daytime circulating thyrotropin, cortisol, and norepinephrine concentrations were increased. Morning postprandial plasma glucose concentrations were also lower compared with controls who slept for 8 h [83]. In a different study, one night of total sleep deprivation increased leptin levels but was not associated with alterations in adiponectin or cortisol levels or of blood pressure, heart rate, or hunger [84].

Reduced sleep quality could negatively impact glucose metabolism even if total sleep time is unchanged. Tasali et al. suppressed SWS in healthy subjects with acoustic stimuli of varying frequencies and intensities such that deep NREM sleep was substituted with shallow NREM sleep, without waking the subject [85]. When deep NREM was suppressed for 3 consecutive nights, insulin sensitivity decreased without an adequate compensatory increase in insulin. Therefore, glucose tolerance was decreased and diabetes risk commensurately increased. The magnitude of the decrease in insulin sensitivity was strongly correlated with the magnitude of the reduction in SWS. These data indicate a role for SWS in maintaining glucose homeostasis. Morning plasma glucose and serum insulin responses were significantly increased following selective SWS suppression in a similarly designed study [86].

Acute or chronic sleep deprivation may induce appetite dysregulation and raise the risk of weight gain, thereby leading to insulin resistance, glucose intolerance, and a concomitant increased risk of diabetes mellitus. In sleep-disordered patients, sleep disruption may result in a cumulative sleep deficit, leading to increased sympathetic nerve activity and elevated evening cortisol. In this scenario insulin resistance, weight gain, and diabetes could be caused [70].

## 6. Impact of Circadian Disruption on Hormones and Metabolism

The melatonin levels of shift workers during night work and daytime sleep were significantly lower compared with those of daytime workers, and morning serum cortisol after work and after sleep were also 24% and 43% lower [87]. Chronic reductions in melatonin and impaired cortisol secretion in night shift workers might exert a carcinogenic effect. However, prolactin levels were not altered during rotating shift work [88].

Night shift workers are characterized by significantly greater postprandial glucose, insulin, and triacylglycerol responses [89]. Several studies indicate that shift working is associated with an increased incidence of metabolic syndrome, obesity, and diabetes [90–92]. Night workers exhibit a greater proportion of body fat mass, lower insulin sensitivity, increased triglycerides, and blunted postmeal ghrelin suppression and xenin release [93]. Xenin, a peptide secreted predominantly in the upper gut, is known to confer a satiating effect. Shift work is associated with increased levels of being overweight and obesity prevalence [94]. In a sleep laboratory study, circadian misalignment was associated with human metabolism. Scheer et al. employed an 11 d forced desynchrony protocol to induce circadian misalignment, all subjects received four isocaloric diet each 28 hour day, following which leptin levels decreased, glucose and insulin increased, cortisol rhythm was reversed, sleep efficiency was reduced, and mean arterial pressure was increased [24]. The study demonstrated the adverse cardiometabolic effects of circadian misalignment, observed acutely during jetlag and chronically during shift work. Sleep deprivation with circadian disruption is viewed as a modifiable risk factor for metabolic disease. Subjects restricted to <5.6 h of sleep/day were characterized by decreased resting metabolic rate and increased plasma glucose concentrations after a meal [95]. Another laboratory study induced sleep deprivation, with and without circadian misalignment; during circadian misalignment, insulin sensitivity increased twofold compared with the nonmisalignment group, and inflammation also increased [96]. Similarly, circadian misalignment was induced using two different light-entrained circadian cycles (21 and 27 h), which altered sleep architecture, dysregulated the HPA-axis, and reduced insulin sensitivity [97]. A recent meta-analysis of the relationship between shift work and diabetes demonstrated an overall effect size of 1.09 [98].

Long-term nightshift working is also associated with decreased total cortisol [99]. In a study of swing shift workers (1 week of nightshift followed by 1 week of dayshift) no reduction in reaction-times or overall health was observed, but cortisol rhythms did not completely normalize even after 4 weeks of holiday [100]. A Japanese study used a 3-year follow-up design to explore the long-term effects of shift work on metabolic syndrome. The odds ratios for metabolic syndrome, of two- and three-shift working patterns, were 1.88 and 0.87, respectively, such that a two-shift working pattern appeared to be a risk factor for metabolic syndrome [101]. In another 4-year follow-up, the relative risk for metabolic syndrome in night shift workers was increased fivefold compared with dayshift workers [102]. In a study by Guo et al., shift work in retired workers was associated with reduced sleep quality, diabetes, and hypertension. Shift work might be associated with long-lasting negative health effects, even after its cessation [103].

In various animal models, circadian disturbances cause metabolic problems. The “night work” experimental model was applied to rats subjected to 8 h forced activity during rest and active phases, which disrupted clock and metabolic gene rhythms. The daily peak of PER1, BMAL1, and clock rhythms was inverted while PER2 rhythm was lost in the liver; NAMPT and PPAR*α* genes, involved in metabolism, lost their rhythm and synchrony with clock genes, which could result in metabolic syndrome and obesity [104]. Circadian disturbances provoked by dim lights at night (dLAN) increased body mass, reduced glucose tolerance, and disrupted the timing of food intake in mice [105]. When exposed to dLAN at night, the amplitude of PER1 and PER2 rhythms was reduced in the hypothalamus [106]. In another study, the metabolic disruption induced by dLAN was ameliorated upon its removal [107].

The effects of chronic jet lag were evaluated in mice studies. When mice were exposed to chronic jet lag conditions, the expression of various clock genes such as Per2 and BMAL1 in the liver was dampened, the tumor suppressor gene p53 expression was suppressed, and the cell cycle progression gene c-Myc expression was induced [108]. Another study revealed that chronic jet lag in mice leads to the phase shift of clock genes (Per1, BMAL1, and Per2) and activated expression of p53 and c-Myc in the liver [109].

Feeding pattern has been reported to be a potent zeitgeber for peripheral circadian clocks. Food restriction in mice resets the phase of rhythmic gene expression in the liver, kidney, and heart and resulted in circadian dyssynchrony between central and peripheral clocks [110]. Light phase fed mice gained significantly more weight than mice fed only during the 12 h dark phase and showed higher fat percentage in body composition [111]. In another study, light phase fed mice were associated with larger consumption of meal and calories, tissue-specific alterations in the phases and amplitudes of circadian clock and metabolic genes (greatest phase differences observed in the liver and diminution of amplitudes in epididymal fat, gastrocnemius muscle, and heart), and greater weight gain [112]. Human subjects with nocturnal life (consuming majority of their calories just before overnight sleep) showed weakened association between glucose elevation and insulin secretion, which is likely to be a risk factor of obesity and diabetes [113]. When mice were restricted to be fed in the dark phase, they were protected against obesity, hyperinsulinemia, hepatic steatosis, and inflammation under the high fat diet condition [114]. Tsai et al. reported that mice fed a high fat diet during the dark phase exhibited normal body weight gain and energy balance, increased fatty acid oxidation at whole body, induced fatty acid responsive genes, and improved myocardial contractile function [115]. These data support the hypothesis that ingestion of dietary fat only during the more active/awake period allows adequate metabolic adaptation.

## 7. Conclusion

Evidence suggests that various hormones and metabolic processes are affected by sleep quality and circadian rhythms; such interactions are mediated by numerous clock genes. Hormones such as growth hormone, melatonin, cortisol, leptin, and ghrelin are closely associated with sleep and circadian rhythmicity, and endogenous circadian-regulating mechanisms play an important role in glucose and lipid homeostasis. Sleep disturbances and, particularly, deprivation are associated with an increased risk of obesity, diabetes and insulin insensitivity, and dysregulation of leptin and ghrelin, which negatively impact human health. Circadian disruption, which is typically induced by shift work, may negatively affect health due to impaired glucose and lipid homeostasis, reversed melatonin and cortisol rhythms, dysregulation of leptin and ghrelin, more severe metabolic syndrome, and clock gene rhythm loss. Future research should elucidate the relationship between sleep disturbance and various physical outcomes and identify the optimal therapeutic approach for the resolution of sleep and circadian rhythm disruption through the recovery of clock genes.
